# Book Reviews

**Published:** 1805-01-01

**Authors:** 


### CRITICAL ANALYSIS

OF THE

### RECENT PUBLICATIONS

ON THE

### DIFFERENT BRANCHES OF PHYSIC, SURGERY, AND MEDICAL PHILOSOPHY.

*An Enquiry into the Efficacy of Oxygen in the Cure of Syphilis; to which are subjoined, a few general Observations on its Application in various other Disorders. By* CHARLES PLATT, *Surgeon to the New Finsbury Dispensary, and Member of the Royal College of Surgeons, London.*

THIS work begins with the inquiry, whether gonorrhœa and lues arise from the same poison? in which the author, after recapitulating the arguments of Swediaur, Bell, and Hunter, decides in favour favour of the latter. Mr. Whateley's revived notion of ulcers in the urethra is introduced; and Mr. Platt expresses his suprize at an opinion so inconsistent with experience, an accurate knowledge of the parts, and of the disease. Some practical remarks follow, on the treatment of gonorrhœa virulenta and gleet, which evince the industry and good sense of the author.

We now enter on the history of the substitution of nitrous acid, and afterwards of oxygen, in any form for the mercurial salts, traced from Mr. Scott to Dr. Beddoes, Mr. Watt, Dr. Rollo, and Mr. Cruickshank. After this follow the author's own experiments. Four cases of well marked primary venereal ulcers or chancres are related, in which the nitrous acid was ineffectually tried, and the patients were at last cured by mercury. Two others are subjoined, in which the same consequences followed, from the use of oxygenated muriatic acid, and two more, one of primary, the other of secondary symptoms, in which oxygenated muriate of pot-ash proved equally ineffectual.

The author now enters into the most important part of the work, viz. to show that all this difference of opinion must have arisen from some difference not sufficiently attended to in the local diseases thus treated. He shows how necessary it is to be more accurate in discriminating appearances, before we give credit to remedies. "This interesting inquiry, he observes, was originally instituted by Mr. Hunter, and hath since been prosecuted with much labour and ingenuity by Dr. Adams,* who has thrown a considerable portion of light on a subject hitherto involved in much obscurity." Some long quotations follow from Dr. Adams's work, tending to prove the variety of ulcers to which the genitals have been always liable, as appears by the writings of authors who described them before the venereal disease was known. Dr. Willan's observations on prurigo pudendi, are next introduced, as a further proof of the uncertainty of these local complaints. Lastly, Mr. Hunter's suspicions, "that new poisons are arising daily, in many respects similar to the venereal, though not in all," close the general quotations on this enquiry.

The author now touches on a subject which we cannot help wishing he had omitted altogether or informed himself better. "There is, says he, another disease, which from its occasionally attacking the genitals, and assuming some similarity to syphilis, is frequently confounded with it. In Africa, the West-Indies, and other places, it is known under the name of yaws; in Scotland, is usually termed sivvens or sibbens; and which Mr. Bell and some other writers, consider as a variety or peculiarity of the venereal disease." It is unnecessary to follow our author any further on this subject, as there is reason to believe he is mistaken in his first position. Though sivvens in Scotland is sometimes called yaws, yet

* Vide Adams's Observations on Morbid Poisons, Phagedæna, and Cancer.

yet yaws in Africa and the West-Indies is never called sivvens, and the two diseases are perfectly different in character, and the course each of them pursues. It is very remarkable that Mr. Platt should have fallen into this error, when the authorities to which he refers, are in direct opposition to him. Dr. Gilchrist and Mr. Bell both speak of sivvens as incurable, excepting by mercury, and say nothing concerning the impossibility of the same persons being infected twice in their lives. Mr. Hunter speaks of yaws as a disease which runs through its course, and leaves the patient for ever free from a future susceptibility of the infection. It is true, neither of them describe the diseases they respectively mention with sufficient accuracy. This is still a grand desideratum. In one of our Journals, however, the reader will find that Dr. Adams has received the prize from the London Medical Society, for his description of yaws; and we trust his late journey into Scotland for that purpose, will furnish a satisfactory account of sivvens.

Mr. Platt now attempts an accurate discrimination of the chancre from all the other ulcers on the genitals, with which it has been confounded. His first diagnosis is that hard or thickened edge and base which is peculiar to this disease. His next remark is, that the chancre is attended with little or no pain whilst the phagedænic ulcer is peculiarly painful. This last distinction we apprehend the author intends to be considered as only comparative, for the chancre is always attended with uneasiness, and in some irritable habits with considerable pain. Two valuable cases are subjoined of ulceration in the genitals, of that complexion which is often mistaken for venereal, and which the Author treated as such. But the progress of the diseases, and the remedies to which they yielded, convinced him of his mistake, and induced him to class the symptoms "under that description of diseased action which Dr. Adams has denominated the sloughing phagedæna or *nigrities serpens* of Celsus." This section closes with some judicious observations, strengthened by Lord Bacon and Mr. Hunter, on the indiscriminate love of novelty, and the necessity of accuracy and patience in pursuing every inquiry.

Another division of the work follows, entitled, "Observations on the Pneumatic Doctrine." This contains many judicious remarks, but not much novelty. The reader will, however, find amusement in this recapitulation of the crudities of some modern practitioners, whose language was at one time so decisive. On the whole, we have found much satisfaction from the perusal of this tract, which we recommend to every practitioner who wishes so correct his knowledge, and render his practice at once more interesting and useful. We hope the Author will pursue his inquiries, and doubt not his future performances will be free from those little imperfections which in this we have reluctantly marked.

---

*The*

*The Invalid; with the obvious Means of enjoying Health and long Life.* *By* A NONAGENARIAN, *Editor of the Spiritual Quixote, Columella, Reveries of Solitude, &c.* 8vo. pp. 147. Lond. 1804.

IT is the title only of this little publication that gives it any claim to notice from the medical reader; but the lounger may find some spare minutes agreeably employed in running over a few desultory remarks and precepts of temperance, which always derive an interest in coming from the pen of one who (we suppose) has been enabled through temperance to reach the term of extreme age. The following is a sample of the pleasant garrulity of this second Cornaro.

"But man, as lord of the creation, by his prerogative, falls foul on whatever comes in his way, and ransacks the universe to gratify his voracious appetite; the fowls of the air, the fishes of the sea, the beasts of the forest, with vegetables of every *genus* and every *species;* not only "herbs, which were intended for the use of man," but roots, which seem reserved for the food and the snouts of hogs; nay, even the excrescences of nature, mushrooms, and truffles, indigestible substances; which if they were ever intended to be eaten, it must probably have been by the inhabitants of the infernal regions.

"If temperance, however, regulated our use of these various articles of food with which Providence indulges us; if we killed the animals without cruelty, and cooked them with plainness and simplicity, they might be what Providence intended them, instead of what we too often make them; a blessing and not a curse: but when we torture them in taking away their lives, as we often do, and scarify and carbonade, and bedevil their flesh not only with pepper and salt, as we do the gizzard of a turkey, but adding a little *nutmeg*, a little *cinnamon*, a blade of *mace*, with chalot and onions, &c. and eat it with oil, vinegar, or mustard; such an heterogenous mixture, instead of producing a lacteous chyle, flowing through the alimentary canal, like the gentle stream of Arno, must become a caustic fluid, rushing like the fiery torrent of Vesuvius, harrowing up and tearing the vessels; or at least generate fevers, calentures, and every disease incident to the human body.

"The luxury of man, however, revenges itself upon his rapacity, and brings forth fever, gouts, and rheumatisms, and all the contents of Pandora's box, which infest the *human species* in every part of the civilized world.

"Some author calls physic and physicians necessary evils; they certainly are so; but we ourselves, by our excess and intemperance, make them necessary. A young extravagant spendthrift considers the law as a nuisance, and bum-bailiffs as his mortal foe; but, let him be more frugal, and a better œconomist, and the evil ceases, and jails and catchpoles become harmless things.

"Sydenham observes, a disease or fever, though inimical to the human body, is nothing more than the effort of Nature to expel the morbific matter, for the health and emolument of the patient. This seems seems to be the merciful provision of Heaven, to counteract the insatiate gluttony of the human race.

"Dryden, in his well-known lines, says, with truth, no doubt, that

> 'The first physicians by debauch were made,
> 'Excess began, and sloth maintains the trade:
> 'The wise for health on exercise depend—

"But, when he adds,

> 'God never made his work for man to *mend*—

"The poet imposes upon himself, as well as on his reader. Instead of *mend* he meant *improve*. A country carpenter could not *improve* a coach or phaeton made by a coach-maker; but if the axletree or wheel were broke, he could *mend* or repair. This is applicable to the physicians.

"The philosopher, Pythagoras, after having travelled over India, on his return settled in Italy, and, thence passing over into Sicily, said, the most remarkable circumstance he had observed in his travels, was a people who made two meals in a day.

"What would this philosopher then say, should he, in a state of transmigration come into England, (in the person of the wandering Jew suppose) where, instead of *praying* seven times a day, as the ancient Jews did, we *eat* seven times a day—we breakfast at ten in the morning, suppose we eat sandwiches or noonchins at three o'clock, dine at six, (I speak of sober people,) drink tea at eight sup at eleven, and if we spend a convivial evening, olives, or anchovies must give a zest to our liquor; if a ball is given, jellies, macaroons, &c. &c.; if hot, something more substantial is expected; and probably the day is concluded, as well as begun, with a second breakfast at five o'clock in the morning."

---

*Elements of Galvanism, in Theory and Practice; with a comprehensive View of its History, from the first Experiments of Galvani to the present Time; containing also, practical Directions for constructing the Galvanic Apparatus, and plain systematic Instructions for performing all the various Experiments. Illustrated with a great number of copper-plates.* By C. H. Wilkinson, Lecturer on Galvanism in Soho Square, &c. &c. Two vol. 8vo. pp. 468. London, 1804.

In a very candid and sensible Preface, the author explains his design in the following manner:

"Soon after the important experiments of Galvani were announced, they were repeated and diversified by the most eminent Physiologists in every part of Europe. Their opinions having been diffused through different philosophical publications, the selection and arrangement of such of them as have been deemed worthy of the reader's attention, have been attempted in this present work.

"These materials I have not arranged systematically, but have principally been governed by the era of the original discovery. I have have divided the work into the two epochs which appeared the most interresting; the first consisting of the various discoveries, from the period of Galvani to that of the Voltaic pile; when the second epoch commences, and is carried down to the present time.

"The Historical Details constitute the first volume, and part of the second. The remaining portion I have devoted to the development of that particular theory which I have for some time entertained, and have adhered to the arrangement which I purposely made for the lectures I have given upon this subject.

"I have endeavoured to demonstrate the principles of Galvanism by those of Electricity. As, on this latter subject, I have presumed to entertain opinions contrary to those generally admitted, it has appeared to me absolutely necessary to set out by stating these opinions, before I should enter on the Elements of Galvanism.

"On this account it is that, after having closed the History, I have commenced the Elementary Part by several chapters on Electricity, with a view to the explanation of certain points, on which my ideas will be found to be somewhat novel.

"This work is illustrated by various plates; and such descriptions and explanations are introduced, as will enable every attentive reader to construct his own apparatus, and to repeat with facility any of the experiments. In the medical application of Galvanism, I believe no person in England has been more extensively engaged than myself; the results of my observations materially differ from those of the continental practitioners. In many instances I have derived from its employment the greatest advantage; while, in many others, I have found it productive of no good effect. In some cases, I am even led to think it rather injurious than beneficial.

"When, in the first instance, I entered on the medical application of this principle, I was led by the accounts of Grapengiesser, and other foreign physicians, to entertain the most sanguine hopes that it would prove one of the most active remedies we possess. Experience has taught me that my expectations were too ardent. It is probable, however, that my failure, in the cases in which it has been successfully employed abroad, may have originated from some circumstances of which at present I am not aware. I therefore trust that the statement I have made, will not discourage other practitioners from its use, as it is only from the united labours of professional gentlemen that its real utility can be ascertained.

"There are many complaints, in which I have found the principle of Galvanism a most beneficial and powerful stimulant, greatly superior to any effects I ever experienced from electricity. At a first view it may appear contradictory, on a supposition that both the principles are the same; that Galvanism, which is merely a weaker intensity of electricity, should, notwithstanding, prove most active on the animal frame. The reason of this I have attempted to explain; and I am induced to think that the influence of Galvanism solely depends on the important agency it exercises in animal substances."

The division of the historical part of the subject is very judicious, the Voltaic discovery forming a most important æra in the history of Galvanism, and of natural science in general: and the uncommon beauty and brilliance of many of the experiments connected with the Voltaic pile, having remarkably contributed to render this, at present, a favourite branch of natural philosophy.

We may just remark, that the author's title, *Elements of Galvanism*, does not precisely reach the character of the work; at least two-thirds of it being a *history* of the experiments and opinions of those who have most distinguished themselves in these inquiries; were the present work merely elementary, much of this interesting part would have been superfluous, and a different and more select arrangement would have been required.

The first chapter is deservedly occupied by the discoveries of Galvani, with a short and interesting sketch of his philosophical life, extracted from the Memoirs of the Medical Society of Emulation at Paris. The accident which first led to the detection of that principle in animals, which has since been termed animal electricity, or, commemorating the great discoverer, *Galvanism*, is thus related.

"Galvani being one evening in his laboratory, where he was employed in making experiments in the presence of a party of his friends, several frogs, which had been skinned, and were destined for soup, happened accidentally to be placed on a table, on which was also an electrical machine; between the conductor and the frogs there was a certain space. One of the company who assisted at the experiments, having brought, unintentionally, the point of a knife into contact with the internal nerves of the thigh of one of these animals, the muscles of the limbs were instantly and powerfully convulsed. The wife of Galvani being present, was struck by the novelty of this phenomenon, between which and the disengagement of an electrical spark, she fancied that there was an agreement in point of time. On her making this observation to her husband, he resolved to ascertain the truth of so extraordinary a fact, and accordingly brought the point of a scalpel, or dissecting knife, in contact with the crural nerves of one of the frogs, at the same time that a spark was drawn from the electrical machine. The result was, that the same contractions were manifested. As it was possible that they might have been owing to the simple contact of the scalpel, which might serve as a stimulant, rather than to the disengagement of the spark, Galvani, to clear up this doubt, touched the same nerves of several of the frogs, while the electrical machine was in a state of rest. The contractions which had heretofore been observed did not ensue."

The theory suggested to this acute physiologist by these and similar phenomena, and by a comparison of them with the effects produced by electricity, has been the subject of much controversy; sy; but its ingenuity, and the reputation of the inventor, give it a claim to be commemorated. Mr. W. gives it in the following terms.

"The nerves," observes Galvani, "which are distributed in the different parts of the muscular system, and which receive or transmit the electrical fluid, have all of them a common origin in the cerebral organ; and it is not probable that these nerves, the structure of which is so varied in the universal economy of animals, should be the secretory organs of an homogeneous fluid, such as the one destined to excite the muscular contractions." He consequently supposes, in the first place, that all animals are endued with an inherent electricity appropriate to their economy, which electricity, secreted by the brain, resides especially in the nerves, by which it is communicated to every part of the body. And, secondly, that the principle reservoirs of this electricity, which he calls *animal*, are the muscles, each fibre of which ought to be considered as having two surfaces, and as possessing on this account, both a positive and a negative electricity, at the same time that it, in a manner, represents a small Leyden phial, the nerves with which it is provided being the conductors.

"The mechanism of the various movements is explained by Galvani in the following manner: The electrical fluid is drawn and attracted from the interior of the muscles into the nerves, from whence it afterwards passes to the external surface of the muscles; insomuch, that each discharge of this description of electrical phial is followed by a muscular contraction, which is the effect of the *stimulus* produced by the electricity. He was strengthened in this conjecture by the perfect analogy he fancied he could perceive between the phenomena of the Leyden phial in electricity, and the muscular contractions. However this parallel may have been since weakened by a few isolated facts, or rather, by the conclusions which have been drawn from them, it is certain that there is a striking similitude in the principal points."

It would be foreign from our purpose to attempt an abstract of the contents of the mere narrative part of Mr. Wilkinson's work; a moderate compass would not render it intelligible; and in this part, the author has only the merit of an industrious compiler, the experiments of Valli, Vassali, Volta, Humboldt, Fowler, &c. being duly detailed, and generally in the very words of the respective authors. Mr. W. clearly shews that he has studied the subject very extensively; and the portions that he selects of the different authors who have written on this science, are well calculated to give, in a moderate space, their leading facts and opinions.

We were particularly pleased with the concluding chapter of the first volume, on the application of galvanism to medicine; in which Mr. W. has selected the most curious parts of the very interesting ideas of Galvani, Grapengeiser, and, above all, Humboldt, on this untried and important subject. The reader will see with what ingenuity Galvani has attempted to transfer to the animal economy,

 phenomena phenomena observed in the electric machine; with what judgment Humboldt points out the particular classes of diseases, to the relief of which galvanism offers the fairest prospects, and how much remains to be done in the investigation of this branch of materia medica. Convulsive diseases, those that are generally supposed to depend more peculiarly upon morbid changes of the nervous system, (exclusive of external injury) have naturally occupied the first attention of these medical philosophers. If the success attending these first efforts has not corresponded with the sanguine hopes entertained, and has thereby entirely damped the ardour of pursuit in some zealous followers of this curious branch of natural philosophy, the sensible observations of Humboldt may be of service in keeping up its due importance with the public. Mr. Wilkinson thus details them.

"Humboldt's communication on the application of Galvanism to practical medicine, was addressed to M. Loder. After five years of constant researches into the laws by which the phenomena of Galvanism are governed, and into those which relate to the irritability of the nervous and muscular fibres, this celebrated physiologist was certainly enabled to decide on the application of these phenomena to practical medicine. After having agreed with M. Loder, that the principal advantage of Galvanic discoveries, consists less in its direct application to the derangements of the animal economy, than in the light it may throw on the nature of the nerves, and on the energy with which they are endued, he proceeds to observe that, in an age like the present, when a slow and progressive progress in the sciences is considered as a stationary state; in an age, in short, when a desire is manifested to collect the fruits before the flowers are formed, the experiments which have, from the moment they were conceived and made, promised a speedy and immediate application to the healing art, cannot have failed to attract the particular attention of the public, by whom such an application was in a manner anticipated.

"It is thus that galvanism has been occasionally recommended as the criterion of death, and at other times as a powerful and salutary stimulus in the diseases of the nerves. But, when an idea was in the first instance formed, of the electric fluid and its properties, was it not immediately thought that, in its action on the human body, it would supply a remedy for all the diseases to which it is subject? In the same way, after having noticed the galvanic phenomena, it has been latterly apprehended that two pieces of metal, employed in a particular way, would, as it were by enchantment, recall to life those who have perished by asphyxy, restore sight to the blind, re-establish, in all cases of paralysis, the use of the limbs, and, in a word, produce more salutary effects than the Faculty have been enabled to obtain from the multitude of chemical, mechanical, and other remedies, which have been employed for so many ages.

"Our physiologist, who is not an enthusiast on this subject, examines amines cooly and without prejudice, what the medical science has a right to expect from galvanism, and subjects to a rigorous analysis the opinions propounded on this subject by the most distinguished naturalists. 'Let it not be forgotten,' he observes, 'that the science which is under our consideration, is still in its infancy, notwithstanding the time that has elapsed since its discovery. On the first occasion that soap-bubbles filled with hydrogen gas were observed to rise rapidly towards the ceiling of an apartment, the spectators were far from imagining that this phenomenon would furnish man with the means of elevating himself in the air, and of hovering with security over the ocean itself."

( To be continued. )

---

*A Practical Treatise on the superior Efficacy and Safety of Stizolobium or Cowhage, (the Dolichos Pruriens of Linnæus) internally administered in Diseases occasioned by Worms; wherein are exhibited, a concise Statement of the Symptoms of the Disease, and the Uncertainty of most other Vermifuges now in Use. To which are added, Observations on some other indigenous Anthelmintics of the West Indies; and several Cases not published in any of the former Editions.* By WILLIAM CHAMBERLAINE, Member of the Royal College of Surgeons, London, &c. &c. 12mo. pp. 134. London, 1804.

THE title of this little pamphlet announcing a ninth edition, no analysis of its contents appears to be required. The Author has now, very judiciously, prefixed Dr. Hooper's neat and accurate description of the different species of worms infesting the human body, first published in the second volume of the Memoirs of the Medical Society.

The following practical directions for the exhibition of the Cowhage may be useful, as they are obviously dictated by much experience.

" My usual way of preparing and administering the Cowhage, is in the form of an electuary, with honey, molasses, or syrup of a thick consistence. Formerly I was not in the habit of observing any exact proportion of the quantity of the setæ; but as, since the publication of the former editions of this Treatise, the demand for it has increased beyond my expectations, I have found it necessary to adopt certain formulæ for ascertaining the proportions; which proportions, although I find them in general to answer very well, I nevertheless, in some particular cases, find it necessary to vary; for there never yet existed any general rule, to which some exception or exceptions could not be found. After repeated trials and experiments, in the course of five and twenty years, (during which period I have been in the constant habit of exhibiting the Cowhage as an anthelmintic) made with a view of finding out the best vehicle for this substance, I cannot say that I have found any less exceptionable than the good old vehicle, common treacle, such as is to be had at every grocer's. I have tried conserves, but children ren cannot be prevailed on so readily to take them. Honey would not be an incommodious vehicle, but it is not with every stomach honey will agree; for it is well known, that in some constitutions, violent colicky complaints are brought on by the smallest quantity of honey, or even by drinking any kind of vinous liquor in which honey enters as an ingredient: and there are these advantages in treacle. First, that every body knows what it is. Secondly, there are few children who do not like it. Thirdly, it is not apt to be spoiled, or to ferment, unless kept in too warm a place. And, lastly, it is gently aperient, and, in that view, an auxillary to the principal ingredient. But if, from a dislike of treacle, some other vehicle would be preferred, raspberry jam or currant jelly will prove very good substitutes.

"At the request of some indulgent parents, in order to *cheat* into compliance such of their children as could not be prevailed on to take any thing that has the appearance of a *medicine*, I was induced to turn in my mind how to exhibit the Cowhage in the form of a lozenge; and after some trials, succeeded in fixing on a formula that answers pretty well. It consists of a due proportion of things extremely simple: sugar, Indian arrow root, and gum tragacanth, but no efficient article except the Cowhage; unless in some few instances of private practice, or at the desire of the medical practitioner who attends in the family of the patient.

"But, though I have had many communications of the good effects of the lozenges, I cannot say I place so much dependance on them, or recommend them in my own practice, (unless where I meet with refractory and spoiled children, that are masters and mistresses over their mamma's) as I find the simple electuary, made with nothing but Cowhage and treacle, answer every purpose.

"Of this electuary, a tea-spoonful is in general found to be a sufficient dose for children, from infancy to the age of six or eight; from thence to fourteen, a desert-spoonful is found to answer well; and for all above that age, a table-spoonful. Formerly, I thought it might be sufficient if taken once a day; but experience has shewn me, that it answers better when taken twice; viz. at night, going to bed, and in the morning AN HOUR BEFORE BREAKFAST; and though little or no previous medicine is necessary, yet it is *generally* found to operate more effectually where a *gentle emetic* (provided nothing forbids it) has been premised.

"The Cowhage, after being begun upon, is to be continued for three or four days; after which, some brisk purgative, such as jalap, or infusion of senna, or, in short, whatever purging medicine is known to agree best with the patient, is to be taken; which will in general bring away the worms, if there be any. Afterwards, the Cowhage is to be continued as long as there may seem occasion; repeating the purgative at intervals of three or four days."

*Account*

